# High-Intensity Interval Training Among Heart Failure Patients and Heart Transplant Recipients: A Systematic Review

**DOI:** 10.7759/cureus.21333

**Published:** 2022-01-17

**Authors:** Ann Kashmer D Yu, Fatma Kilic, Raghav Dhawan, Rubani Sidhu, Shahd E Elazrag, Manaal Bijoora, Supriya Sekhar, Surabhi Makaram Ravinarayan, Lubna Mohammed

**Affiliations:** 1 Internal Medicine, California Institute of Behavioral Neurosciences & Psychology, Fairfield, USA; 2 Plastic and Reconstructive Surgery, California Institute of Behavioral Neurosciences & Psychology, Fairfield, USA; 3 Anesthesiology, California Institute of Behavioral Neurosciences & Psychology, Fairfield, USA; 4 Psychiatry, California Institute of Behavioral Neurosciences & Psychology, Fairfield, USA; 5 Emergency Medicine, California Institute of Behavioral Neurosciences & Psychology, Fairfield, USA; 6 Paediatrics, California Institute of Behavioral Neurosciences & Psychology, Fairfield, USA

**Keywords:** exercise, heart transplantation, heart failure, cardiac rehabilitation, high-intensity interval training

## Abstract

High-intensity interval training (HIIT), an exercise training modality of cardiac rehabilitation, has shown growing evidence of improving cardiovascular patients' prognosis and health outcomes. This study aimed to identify and summarize the effects of HIIT in heart failure (HF) patients, heart transplantation (HTx) recipients, and HF patients before and after HTx. This systematic review was based on the Preferred Reporting Items for Systematic Reviews and Meta-Analyses (PRISMA) guidelines. For the past five years, a systematic search was done using PubMed, PubMed Central, Cochrane, Google Scholar, and ScienceDirect databases on September 15, 2021. Studies were selected based on the following predefined eligibility criteria: English-language randomized controlled trials (RCTs), observational studies, systematic reviews, and meta-analyses, which included HF patients and HTx patients, and assessment of effects HIIT. The relevant data were extracted to a predefined template.

Consequently, quality assessment was done using each study's most commonly used assessment tools. The initial search generated 551 studies. Nine studies were included in the final selection - four RCTs, one cohort, one quasi-experimental study, two systematic reviews with meta-analyses, and one narrative review. HIIT was found to be generally superior or similar with other exercise training on VO_2_ peak, heart rate, LVEF, cardiac biomarkers, vascular function, blood pressure, body composition, and adverse events in HF patients and the aforementioned with QoL among HTx recipients. Data on cardiac remodeling and QoL of HF patients were inconclusive.

## Introduction and background

Cardiovascular diseases (CVDs) remain the leading cause of mortality worldwide. In 2019, around 17.9 million died due to CVDs which denotes 32% of global deaths [1,2]. According to WHO, CVDs include coronary heart disease, cerebrovascular disease, peripheral artery disease, rheumatic heart disease, congenital heart disease, deep vein thrombosis, and pulmonary embolism [1]. Some of these can progress to a clinical syndrome of heart failure (HF), which may serve as their endpoint. Several guidelines in diagnosing HF show relatively different criteria. However, establishing the presence of HF in these guidelines is emphasized for optimum management and prognosis, while pharmacological therapy is considered the leading treatment. In addition, these guidelines recommend preventive strategies to delay the progression of HF [3]. Heart transplantation (HTx) is recommended [4]. The survival rate for either HF or post-HTx has increased over time, and the one-year survival rate is 80-90% and 91%, respectively [4-6]. Despite this, these patients' quality of life (QoL) is below normal than the average individual, and their prognosis may plateau over time, underlining the need for improving evidence-based treatment [5]. 

Exercise capacity measured using maximum peak oxygen (VO_2_ peak) consumption and other factors improving the QoL are generally associated with increased survival and decreased morbidity and mortality in HF and HTx. Thus, these factors were considered and documented [7,8]. At present, different guidelines recommend exercise-based cardiac rehabilitation, especially exercise training, as secondary and tertiary prevention in improving the prognosis of HF patients and HTx recipients [9,10]. From these, moderate continuous exercise (MCT) is considered the most established form of prescribed exercise training due to its well-demonstrated clinical benefits and safety [11]. However, emerging studies show that high-intensity interval training (HIIT) as an exercise modality has shown a similar or more significant impact on outcome measures when used as an adjunct or an alternative to MCT. The HIIT is characterized by interval training at high intensity with near-maximal efforts either at an intensity below VO_2_ peak, peak power output, and peak heart rate. This training includes short-, medium- and long-interval HIITs depending on the duration of each interval. These intervals require supervision among beginners, especially among cardiovascular patients [12].

Although many studies have indicated better HIIT outcomes than MCT or guideline-based exercise, some studies contradict this, which is why HIIT is still cautiously recommended among HF patients and HTx recipients. Thus, there is no universal exercise prescription [2,13,14]. Furthermore, there have been no systematic reviews of HIIT effects for both populations. Perhaps this review can serve as a bridge to highlight the effects of HIIT before and after HTx of HF patients. The investigators thereby seek a more concise and more straightforward direction in determining the best HIIT prescriptive outcomes that can provide the most significant benefits among these patients, especially regarding the QoL and improvement in prognostication. Therefore, this systematic review aims to identify and summarize the effects of HIIT in terms of outcome measures among HF patients and HTx recipients. The exercise training outcomes included in the study are VO_2_ peak, heart rate, pulse oxygen (O2), left ventricular ejection fraction (LVEF), cardiac remodeling, cardiac biomarkers, vascular function, blood pressure, body composition, adverse events, and QoL.

Methods

This systematic review was conducted based on the Preferred Reporting Items for Systematic Reviews and Meta-Analyses (PRISMA) 2020 guidelines [15]. 

*Eligibility Criteria* 

The studies were selected based on the Participants, Intervention, and Outcomes (PIO) elements: Participants, HF, or HTx patients, or HF before and after HTx; Intervention, HIIT alone or with MCT; and Outcome, any exercise training outcome measure. In addition, additional inclusion and exclusion criteria were added: Inclusion, English-language, Free Full-Text articles published within the last five years, randomized controlled trials (RCTs), observational studies, systematic reviews, and meta-analyses; Exclusion, case reports, case studies, and editorials. 

Databases and Search Strategy

The search was conducted systematically using PubMed, PubMed Central (PMC), Cochrane, Google Scholar, and ScienceDirect databases. The last date of the search for all databases was September 15, 2021. The field search used in the process were selected based on the keywords used in the previous literature and through Medical Subject Headings (Mesh), depending on the database used, as seen in Table 1.

**Table 1:** The strategy of the bibliographic search in databases with their corresponding filters. PMC - PubMed Central

Databases	Keywords	Search strategy	Filters	Search results
PubMed	High-intensity interval training, High intensity intermittent exercise, Interval training, Exercise, Exercise tolerance Cardiac rehabilitation, Cardiac regimen, Cardiac rehab, Cardiac care Heart failure, Cardiac failure, Heart decompensation, Congestive heart failure, Left heart failure	#1 High-intensity interval training OR High intensity intermittent exercise OR Interval training OR Exercise OR Exercise tolerance OR ( "High-Intensity Interval Training/adverse effects"[Mesh] OR "High-Intensity Interval Training/therapeutic use"[Mesh] )#2 Cardiac rehabilitation OR Cardiac regimen OR Cardiac rehab OR Cardiac care OR ( "Cardiac Rehabilitation/adverse effects"[Mesh] OR "Cardiac Rehabilitation/therapeutic use"[Mesh] OR "Cardiac Rehabilitation/therapy"[Mesh] ) #3 Heart failure OR Cardiac failure OR Heart decompensation OR Congestive heart failure OR Left heart failure OR ( "Heart Failure/prevention and control"[Mesh] OR "Heart Failure/rehabilitation"[Mesh] OR "Heart Failure/therapy"[Mesh] ) #4 Heart transplant OR Cardiac transplant OR Heart transplantation OR ( "Heart Transplantation/rehabilitation"[Mesh] OR "Heart Transplantation/therapeutic use"[Mesh] OR "Heart Transplantation/therapy"[Mesh] ) #5 #1 AND #2 AND #3 AND #4 #6 High intensity interval training OR High intensity intermittent exercise OR Interval training OR Exercise OR Exercise tolerance OR Exercise capacity OR ( "High-Intensity Interval Training/adverse effects"[Majr] OR "High-Intensity Interval Training/therapeutic use"[Majr] ) #7 "Heart failure" OR "Cardiac failure" OR "Heart decompensation" OR "Congestive heart failure" OR "Left heart failure" OR ( "Heart Failure/prevention and control"[Mesh] OR "Heart Failure/rehabilitation"[Mesh] OR "Heart Failure/therapy"[Mesh] ) #8 "Heart transplant" OR "Cardiac transplant" OR "Heart transplantation" OR ( "Heart Transplantation/rehabilitation"[Mesh] OR "Heart Transplantation/therapeutic use"[Mesh] OR "Heart Transplantation/therapy"[Mesh] ) #9 #6 AND #2 AND #7 AND #8 #10 #6 AND #2 AND #8 #11 #6 AND #7 AND #8 #12 #5 OR #9 OR #10 OR #11 – 1,253	Last Five Years, Free Full Text	163
PMC	High-intensity interval training Heart failure Heart transplant Cardiac rehabilitation	#1 High-intensity interval training[Title] #2 Heart failure #3 Heart transplant #4 Cardiac rehabilitation #5 #1 AND #2 AND #3 AND #4 #6 #1 AND #2 AND #4 #7 #1 AND #2 AND #3 #8 #5 AND #6 AND #7 – 102	Open Access, Five Years	72
Cochrane Library	High-intensity interval training Heart failure Heart transplant Cardiac rehabilitation	#1 MeSH descriptor: [High-Intensity Interval Training] explode all trees #2 MeSH descriptor: [Heart Failure] explode all trees #3 MeSH descriptor: [Heart Transplantation] explode all trees #4 MeSH descriptor: [Cardiac Rehabilitation] explode all trees #5 #1 AND #2 AND #3 and #4 #6 #1 AND #2 AND #3 #7 #1 AND #2 #8 #1 AND #3 #9 #1 AND #3 AND #4 #10 #1 AND #2 AND #4 #11 #7 OR #8 OR #9 OR #10 – 19	September 13, 2016 to September 15, 2021	19
ScienceDirect	High intensity interval training Heart failure Heart transplant Cardiac rehabilitation	High intensity interval training AND Cardiac rehabilitation AND Heart failure AND Heart transplant – 1,119	2016-2021, Review Articles, Research Articles, Medicine and Dentistry	120
Google Scholar	High intensity interval training Heart failure Heart transplant Cardiac rehabilitation	"high intensity interval training" AND "heart failure" AND "heart transplantation" AND "cardiac rehabilitation" – 278	2016-2021	177

All references were grouped and alphabetized using Microsoft Excel 2021 for duplicate removal. The records were initially reviewed based on the titles and abstracts, excluding irrelevant studies. After reviewing, a retrieval of the full-text articles followed this. Study protocols were excluded due to the lack of analyses which is needed in this review. Because of the few systematic reviews, meta-analyses, and narrative reviews in the area, the investigators elected not to exclude them in the study.

Risk of Bias in Individual Studies

The full articles remaining were assessed for quality assessment and risk of bias using tools depending on the study type: RCTs, Cochrane Collaboration Risk of Bias Tool (CCRBT); Cohort Studies, Newcastle Ottawa Scale (NOS); Quasi-experimental Studies, Joanna Briggs Institute (JBI) Critical Appraisal Checklist; Systematic reviews and Meta-analyses, Assessment of Multiple Systematic Reviews 2 (AMSTAR 2); and Narrative reviews, Scale for the Assessment of Narrative Review Articles 2 (SANRA 2) [16-20]. Each assessment tool had its criteria and different scoring. A point is given when a tool scores "LOW RISK," "YES," and "PARTIAL YES," or "1". Two points are given when "2" is indicated. A score of at least 70% for each assessment tool was accepted (Table 2).

**Table 2:** Quality assessment of each type of study. CCRBT - Cochrane Collaboration Risk of Bias Tool, NOS - Newcastle Ottawa Scale, JBI - Joanna Briggs Institute, AMSTAR 2 - Assessment of Multiple Systematic Reviews 2, SANRA 2 - Scale for the Assessment of Narrative Review Articles 2, RCTs - Randomized controlled trials, RoB - Risk of bias

Quality assessment tool	Type of study	Items & their characteristics	Total score	Accepted score (>70%)	Accepted studies
CCRBT [16]	RCTs	Seven items: random sequence generation and allocation concealment (selection bias), selective outcome reporting (reporting bias), other sources of bias, blinding of participants and personnel (performance bias), blinding of outcome assessment (detection bias), and incomplete outcome data (attrition bias). Bias assessed as LOW RISK, HIGH RISK or UNCLEAR.	7	5	Ellingsen et al. 2017 [14] Nytrøen et al. 2019 [21] Besnier et al. 2019 [22] Mueller et al. 2021 [13]
NOS [17]	Cohort	Eight items: (1) Representativeness of the exposed cohort (2) Selection of the non-exposed cohort (3) Ascertainment of exposure (4) Demonstration that outcome of interest was not present at the start of study (5) Comparability of cohorts on the basis of the design or analysis* (6). Assessment of outcome (7) Was follow-up long enough for outcomes to occur (8) Adequacy of follow-up of cohorts Scoring was done by placing a point on each category. Scored as 0, 1, 2. * Maximum of two points are allotted in this category.	8	6	Hsu et al. 2019 [23]
JBI [18]	Quasi-experimental	Nine items: (1) Is it clear in the study what is the ‘cause’ and what is the ‘effect’? (2) Were the participants included in any comparisons similar? (3) Were the participants included in any comparisons receiving similar treatment/care, other than the exposure or intervention of interest? (4) Was there a control group? (5) Were there multiple measurements of the outcome both pre and post the intervention/exposure? (6) Was follow up complete and if not, were differences between groups in terms of their follow up adequately described and analyzed? (7) Were the outcomes of participants included in any comparisons measured in the same way? (8) Were outcomes measured in a reliable way? (9) Was appropriate statistical analysis used? Scored as YES, NO, UNCLEAR or NO ACCEPTABLE.	9	7	Lima et al. 2018 [24]
AMSTAR 2 [19]	Systematic review, Meta-analysis	Sixteen items: (1) Did the research questions and inclusion criteria for the review include the components of PICO? (2) Did the report of the review contain an explicit statement that the review methods were established prior to the conduct of the review and did the report justify any significant deviations from the protocol? (3) Did the review authors explain their selection of the study designs for inclusion in the review? (4) Did the review authors use a comprehensive literature search strategy? (5) Did the review authors perform study selection in duplicate? (6) Did the review authors perform data extraction in duplicate? (7) Did the review authors provide a list of excluded studies and justify the exclusions? (8) Did the review authors describe the included studies in adequate detail? (9) Did the review authors use a satisfactory technique for assessing the risk of bias (RoB) in individual studies that were included in the review? (10) Did the review authors report on the sources of funding for the studies included in the review? (11) If meta-analysis was justified did the review authors use appropriate methods for statistical combination of results? (12) If meta-analysis was performed did the review authors assess the potential impact of RoB in individual studies on the results of the meta-analysis or other evidence synthesis? (13) Did the review authors account for RoB in individual studies when interpreting/ discussing the results of the review? (14) Did the review authors provide a satisfactory explanation for, and discussion of, any heterogeneity observed in the results of the review? (15) If they performed quantitative synthesis did the review authors carry out an adequate investigation of publication bias (small study bias) and discuss its likely impact on the results of the review? (16) Did the review authors report any potential sources of conflict of interest, including any funding they received for conducting the review? Scored as YES or NO. Partial Yes was considered as a point.	16	12	Xie et al. 2017 [2] Perrier-Melo et al. 2018 [25]
SANRA 2 [20]	Narrative review	Six items: justification of the article’s importance to the readership, statement of concrete aims or formulation of questions, description of the literature search, referencing, scientific reason, and appropriate presentation of data. Scored as 0, 1 or 2.	12	9	Dun et al. 2019 [12]

Data Collection, Items, and Analysis

Because of the inter-variability between studies, such as heterogeneity of participants, interventions, and outcome measures, this systematic review describes these trials and reviews based on their outcomes, applicability, and limitations on a narrative synthesis rather than on conducting a meta-analysis. Full articles were read, analyzed, and tabulated into (1) clinical trials and observational studies, (2) and reviews. The items gathered from each study included first author-year, study type, disease, inclusion and exclusion criteria, key findings, and funding sources. For clinical trials and observational studies, the exercise protocol, sample size, and demographic characteristics of study participants were added. The reviews included the number and type of studies and total participants and range. 

Outcome Assessment

Studies were grouped according to participants - HF patients or HTx recipients to synthesize the outcomes. In addition, any outcome measure (positive or negative) from the exercise training emphasized in the studies was also included in the review. VO_2 _peak, heart rate, LVEF (%), cardiac remodeling, cardiac biomarkers, vascular function, blood pressure, body composition, adverse events, and QoL. Two independent investigators did data collection, selection, assessment, and analyses in each step. If there was a contradicting result regarding an article's eligibility, its full text was assessed by consensus within the group.

Results

Study Selection and Quality Assessment

**Figure 1:**
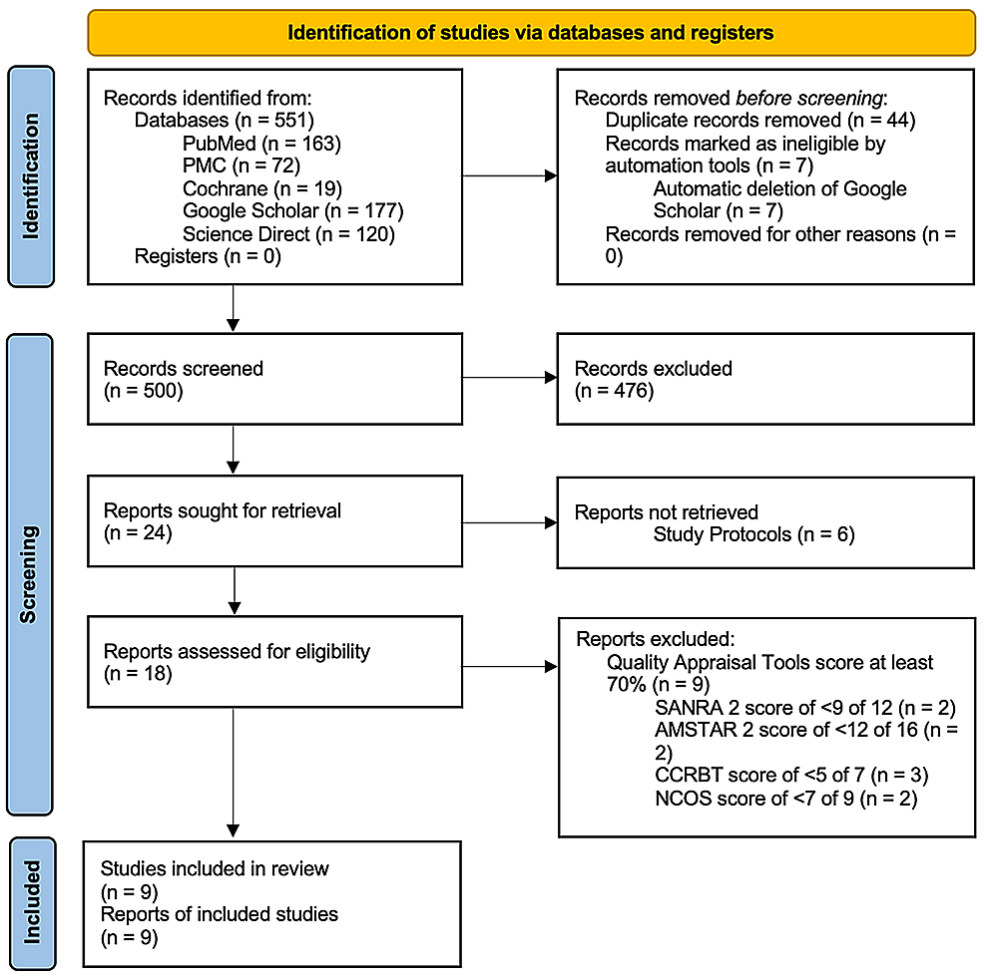
Flow chart of the study search selection. CCRBT - Cochrane Collaboration Risk of Bias Tool, NOS - Newcastle Ottawa Scale, AMSTAR 2 - Assessment of Multiple Systematic Reviews 2, SANRA 2 - Scale for the Assessment of Narrative Review Articles 2

In the database search, there were 551 potentially relevant titles. Seven titles were automatically deleted in Google Scholar. Removal of duplicates was also done with 500 records retained. After duplicate removal, 24 articles remained when the titles and abstracts of these records were screened based on this review's PIO elements and eligibility criteria. These articles were retrieved, and six study protocols were excluded. Finally, a quality assessment for each article was done, and nine studies with a score of greater than 70% were accepted in the review. These were four RCTs, one cohort, one quasi-experimental study, two systematic reviews with meta-analyses, and one narrative review. No other resources were added. A flow diagram showing the screening process and study selection is presented in Figure 1.

Tables 3-7 show how each study was evaluated using the corresponding quality assessments tool for each type of study. All RCTs assessed in the review used the CCRBT and had a "LOW RISK" bias for random sequence generation. However, one of the accepted RCTs had a "HIGH RISK" bias in blinding participants and personnel. This study was still included because of the nature of the intervention and a score of five out of seven. Table 3 below presents these findings.

**Table 3:** Risk of bias summary of randomized controlled trials by review authors. LR - Low risk, UN - Unclear, HR - High risk

First author, Year	Random sequence generation	Allocation concealment	Selective outcome reporting	Other bias	Blinding of participants and personnel
Ellingsen et al. 2017 [14]	LR	LR	UN	LR	UN
Abdelhalem et al. 2018 [26]	LR	UN	LR	LR	UN
Nytrøen et al. 2019 [21]	LR	LR	UN	LR	LR
Besnier et al. 2019 [22]	LR	LR	LR	LR	LR
Nytrøen et al. 2020 [27]	LR	UN	UN	LR	UN

Table 4 shows all cohort studies assessed using the NOS tool, and the accepted cohort study had a score of “1” for each item. 

**Table 4:** Result summary of coding manual for cohort studies by review authors.

First author, Year	Item 1	Item 2	Item 3	Item 4	Item 5	Item 6	Item 7	Item 8
Hsu et al. 2019 [23]	1	1	1	1	1	1	1	1
Busin et al. 2021 [28]	1	1	0	1	1	1	1	0
Villela et al. 2021 [29]	1	0	1	1	0	1	1	0

The JBI tool was used in assessing the only quasi-experimental study in the review. This study scored seven out of nine, with Items 3 and 4 recorded as "NO" because of different medications used by the participants. Furthermore, there was a control group in the study (Table 5).

**Table 5:** Result summary of critical appraisal for quasi-experimental studies by review authors. Y - Yes, N - No, UN - Unclear, NA - Not applicable

First author, Year	Item 1	Item 2	Item 3	Item 4	Item 5	Item 6	Item 7	Item 8	Item 9
Lima et al. 2018 [24]	Y	Y	N	N	Y	Y	Y	Y	Y

One study is a systematic review, while the rest were systematic reviews with meta-analysis. Upon scoring using AMSTAR 2 tool, two of the accepted reviews had "NO" in Items 2, 12, and 13. One review has "NO" in Item 15, while the other is in Item 10. These discussed heterogeneity and funding sources, respectively, as presented in Table 6.

**Table 6:** Result summary of critical appraisal for systematic reviews and meta-analyses by review authors. Y - Yes, PY - Partial yes, N - No

First author, Year	Item 1	Item 2	Item 3	Item 4	Item 5	Item 6	Item 7	Item 8	Item 9	Item 10	Item 11	Item 12	Item 13	Item 14	Item 15	Item 16
Xie et al. 2017 [2]	Y	N	Y	PY	Y	Y	PY	PY	PY	N	Y	N	N	Y	Y	Y
Perrier-Melo et al. 2018 [25]	Y	N	Y	PY	Y	Y	PY	PY	Y	Y	Y	N	N	Y	N	Y
Wewege et al. 2018 [30]	Y	N	N	PY	Y	Y	PY	PY	PY	N	N	N	Y	Y	N	N
Conceição et al. 2020 [31]	Y	N	N	PY	Y	Y	PY	PY	PY	N	Y	N	N	Y	N	Y

Finally, Table 7 demonstrates the scoring of narrative reviews using the SANRA 2 checklist based on six items. The accepted review scored "0" in the description of the literature search and "1" in the appropriate presentation of data.

**Table 7:** Result summary of quality assessment of narrative reviews by review authors.

First author, Year	Justification of the article's importance for the readership	Statement of concrete aims or formulation of questions	Description of the literature search	Referencing	Scientific reasoning	Appropriate presentation of data
Gayda et al. 2016 [32]	2	1	0	2	2	1
Dun et al. 2019 [12]	2	2	0	2	2	1
Ito 2019 [33]	2	1	0	2	2	1

Study Characteristics

The main characteristics of the clinical trials and observational studies and reviews are shown chronologically in Tables 8-9, respectively. Of the nine studies accepted in the review, seven articles had a population of HF patients, while two focused on HTx recipients. No study had HF patients who underwent HIIT before and after HTx. The studies included 2,511 participants, with 1,175 receiving HIIT intervention alone or as an adjunct, and 1,336 receiving MCT, recommendation of regular exercise (RRE), guideline-based physical activity (GB), or multidisciplinary disease management program (MDP). Two trials and two reviews focused on assigned patients in HIIT or MCT, and one trial for HIIT, MCT, and recommended exercise. In addition, one cohort study compared HIIT + MDP, and the quasi-experimental study assessed HIIT alone. One review also assessed HIIT with usual care. 

There were 723 participants for HF among the RCTs and observational studies and 81 participants for HTx. Of these participants, there was a 0-24% dropout rate in each study. Among these studies, the mean age was 61.06 years (HIIT alone or with MCT= 60.83 years; other interventions= 61.26 years), and 65% were men. Table 8 below shows these findings.

**Table 8:** Main characteristics of the randomized controlled trials and observational studies accepted in the review. RCT - Randomized controlled trial, M - Males, F - Females, NR - Not reported, HF - Heart failure, HFpEF - Heart failure preserved ejection fraction, HFrEF - Heart failure reduced ejection fraction, HTx - Heart transplant, CHF - Congestive heart failure, NYHA - New York Heart Association, CI - Contraindications, LVEF - Left ventricular ejection fraction, HIIT - High-intensity interval training, MCT - Moderate-continuous training, RRE - Recommendation of regular exercise, MDP - Multidisciplinary disease management program, GB - Guideline-based physical activity

First author, Year	Study type	Disease	Inclusion & Exclusion criteria	Exercise	Sample size (Dropouts)	Gender, Age	Training, Frequency, Length & Follow-up	Outcomes & Key Points	Funding sources
Ellingsen et al. 2017 [14]	RCT	HF	I: Symptomatic NYHA class II-III, stable, optimally treated CHF, LVEF <35% at local centers and <40% in labs; E: NR	HIIT	90 (13)	M (63)/ F (14) 65	Treadmill/bicycle; (Total: 38 min/session) Warm-up, four blocks (four minutes of HIIT at 90-95% maximal heart rate separated by three-minute active recovery periods of moderate-intensity, cool-down. Three days/week for 12 weeks. Follow-up after 52 weeks.	There is no significant difference between HIIT and MCT for cardiac remodeling and aerobic capacity.	This study was supported by multiple institutions.
				MCT	85 (20)	M (53)/ F (12) 60	Treadmill/bicycle; Forty-seven minutes at 60-70% maximal heart rate. Three days/week for 12 weeks. Follow-up after 52 weeks.		
				RRE	86 (13)	M (59)/ F (14) 60	Exercise at home based on current recommendations and attend one session every three weeks for 12 weeks. Follow-up after 52 weeks.		
Lima et al. 2018 [24]	Quasi-experimental study	HF (HFpEF)	I: Signs & symptoms of HF, preserved ejection fraction of > 50%, diastolic dysfunction with increased filling pressure; and in the case of E/e’ < 15, at least one diagnostic criterion for HFpEF, 40–75 years, NYHA class I to III, and clinically stable with optimal pharmacological therapy in greater than three months; E: Severe lung disease, moderate-to-severe valvular disease, peripheral arterial disease, autonomic neuropathy, unstable angina, history of stress-induced complex arrhythmias, implantable cardiac electronic devices, cognitive and limiting musculoskeletal problems.	HIIT	16 (0)	M (7)/ F (9) 59	Treadmill; (Total: 36 min/session) Eight minutes warm-up, four blocks (four minutes of HIIT at 85-95% maximal heart rate, 15 to 17 on Borg rating of perceived exertion scale) alternated with three minutes at 60-70% maximal heart rate, 11 to 13 on Borg scale, three minutes cool-down. One session. No follow-up.	A single HIIT session can increase the brachial artery diameter and reduce blood pressure. However, it does not change flow-mediated dilation and diastolic blood pressure.	The study was funded by multiple institutions.
Hsu et al. 2019 [23]	Cohort study	HF (HFpEF, HFrEF)	I: HF patients diagnosed based on Framingham HF diagnostic criteria, stable greater than four weeks; E: ≥ 80 or <20 years old, unable to exercise due to non-cardiac disease, pregnancy, interrupted exercise training during follow-up, lost to follow-up, candidates for cardiac transplantation within six months, uncompensated HF patients, or renal patients with an estimated glomerular ﬁltration rate of <30 mL/min/1.73 m2, absolute CI for cardiopulmonary exercise test, and aerobic activities	HIIT + MDP	101 (0)	M (70)/ F (31) 61.5	Bicycle; Five blocks (Three minutes of HIIT at 80% peak VO_2_) separated by three-min exercise at 40% peak VO_2 _two to three sessions/week for three to four months. Follow-up after 51.2 months.	HIIT increased VO_2_ peak and decreased LVESD. Both of these are associated with improved survival in HF patients. Resting HR was higher in MDP.	This study was supported by grants from multiple institutions.
				MDP	133 (32)	M (74)/ F (27) 62.8	Home-based physical activities. Follow-up after 52 months.		
Nytrøen et al. 2019 [21]	RCT	HTx (3 months after transplantation)	I: Clinically stable, >18 years old, undergoing immunosuppressive therapy, with informed consent, motivated to participate for nine months, should be six to eight weeks post-surgery; E: Patients with cognitive issues and physical disabilities; medical complications, language barriers, contagion; unavailable physical therapist, and transplantation of multiple organs	HIIT	39 (2)	M (28)/ F (9) 50	Ten minutes warm-up, four blocks (two to four minutes of HIIT at 85% to 95% of peak effort (85%–95% of peak HR or ≈81%–93% of VO_2_ peak)), three blocks (three minutes of MCT), five minutes cool-down. Progressively increasing in intensity: (three to six months after HTx) one HIIT session, one resistance training session (core musculature and large muscle groups), and one combined session per week; (six to nine months after HTx) two HIIT sessions and one resistance training session (the last with or without supervision) per week; and (last two to three months) three HIIT sessions per week. Nine months. Follow-up after one year from HTx.	In comparison with MCT, HIIT has a clinically more significant improvement in VO_2_ peak values (25% vs. 15%), anaerobic threshold, peak expiratory flow, and muscular exercise capacity.	This study was supported by grants from multiple institutions.
				MCT	42 (1)	M (29)/ F (12) 48	Ten minutes warm-up, 25 min exercise (60-80% peak effort), five minutes cool-down. Nine months. Follow-up after one year from HTx.		
Besnier et al. 2019 [22]	RCT	HF	I: Stable CHF NYHA class I to III, LVEF < 45% for greater than six months, optimal pharmacological treatment greater than six weeks, and ability to perform cardiopulmonary exercise test; E: Exercise training ﬁxed-rate pacemaker with HR limits set less than target HR, major cardiovascular event or procedure within the three months, chronic atrial ﬁbrillation; HF secondary to signiﬁcant uncorrected primary valve disease, congenital heart disease or obstructive cardiomyopathy	HIIT	16 (0)	M (11)/ F (5) 59.5	Five minutes warm-up, two blocks (eight minutes of HIIT alternating between 30 sec at 100% of peak power output and 30 sec of passive recovery) separated by four minutes of passive recovery, five minutes of cool-down at 30% of peak power output. Five days/week for 3.5 weeks. No follow-up.	HIIT was superior to MICT in enhancing parasympathetic tone and peak oxygen uptake. However, there is no association between each of the outcomes.	No special grants were received in any sector.
				MCT	16 (1)	M (11)/ F (4) 59	Cycling; Five minutes warm-up, 30 min at 60% of peak power output, five minutes of cool-down at 30% of peak power output. Five days/week for 3.5 weeks. No follow-up.		
Mueller et al. 2021 [13]	RCT	HF (HFpEF)	I: Signs & symptoms of HFpEF, NYHA class II-III, LVEF of >50%, and elevated estimated LV filling pressure or E/e′ medial of eight or greater with elevated natriuretic peptides; E: NR	HIIT	60 (4)	M (17)/ F (41) 70	(Total: 38 min/ session) 10-minute warm-up, four blocks (four minutes of HIIT at 80%-90% of heart rate reserve, interspaced by three minutes of active recovery), three times per week for 12 months (three months clinic, then nine months supervised via telemedicine at home). Follow-up after three months.	In HFpEF, there is no statistical difference in the change of peak VO_2_ between HIIT and MCT. The study does not support either HITT or MCT compared with GB for patients with HFpEF.	Grants were received from multiple sources.
				MCT	60 (5)	M (23)/ F (35) 70	Five times per week for 40 min (35%-50% of heart rate reserve) in 12 months (three months clinic, then nine months supervised via telemedicine at home). Follow-up after three months.		
				GB	60 (5)	M (19)/ F (41) 69	One-time advice on physical activity according to guidelines for 12 months (three months clinic, then nine months supervised via telemedicine at home). Follow-up after three months.		

There were 13, three, and 21 RCTs in the three reviews, respectively. These reviews included 1,589 HF and 118 participants for HTx and provided clear inclusion and exclusion criteria for HF and HTx participants. However, the process of patient selection, whether they were all-comers or volunteers were generally not stated. Most studies involving patients with HF included patients with functional classiﬁcation up to New York Heart Association Class III and were clinically stable. Funding sources differed with each study. Table 9 presents these findings.

**Table 9:** Main characteristics of the narrative reviews, systematic reviews, and meta-analysis accepted in the review. HF - Heart failure, HTx - Heart transplant, RCTs - Randomized controlled trials, HIIT - High-intensity interval training, MCT - Moderate-continuous training, NR - Not reported

First author, Year	Study type	Disease	No. & Type of included Studies	Total participants, Range	Inclusion & Exclusion criteria	Outcomes & Key points	Funding sources
Xie et al. 2017 [2]	Systematic review with Meta-analysis	HF	21 RCTs	HIIT (363)/ MCT (377) 7-85/ 6-89	I: Only full-text studies in English, articles comparing outcomes between HIIT as the interval group and MCT as the control group, with rhythmic aerobic exercise programs lasting greater than four weeks; at least one cardiorespiratory exercise training outcome in cardiac patients; E: Reviews, cases reports, editorials, communications, and reports without sufficient data	HIIT improved aerobic capacity more effectively than MCT in cardiac patients.	NR
Perrier-Melo et al. 2018 [25]	Systematic review with Meta-analysis	HTx	3 RCTs	HIIT (60)/ Control (58) 14-24/ 13-24	I: RCTs assessing peak VO_2_ and/or HR peak as the primary outcome; participants exclusively of HTx recipients; studies assessing the HIIT effect; and studies with an intervention greater than weeks; E: Studies without a control group, with acute analysis, and case studies	HIIT improved VO_2_ peak, heart rate, and blood pressure in eight to twelve weeks of intervention among HTx recipients.	No external funding sources were received in this study.
Dun et al. 2019 [12]	Narrative review	HF	13 RCTs	HIIT (430)/ MCT (419) 9-100/ 6-100	NR	Both subjective and objective measures should in prescribing HIIT intensity.	NR

The HIIT protocol for every study varied from two to five blocks (two to eight minutes of HIIT) with five to 10-minute warm-ups. These protocols were achieved in varying measurements such as maximal heart rate, peak power output, maximal VO_2_ peak and subjective measurements (Borg rating), and kinds of workouts like bicycle, treadmill, or both. Other interventions were MCT, RRE, MDP, and GB, either supervised, individually advised, or combined.

Outcomes

The outcomes were divided into two populations - HF and HTx. Two studies discussed HTx, while the rest elaborated more on HF. Of the nine studies, eight studies discussed VO_2_ peak, six studies for heart rate, four for VEF, four for cardiac remodeling, five for cardiac biomarkers, five for vascular function, three for blood pressure, four for body composition, seven for adverse events and four for quality of life. Table 10 shows the outcomes of the accepted studies in this review.

**Table 10:** Outcomes addressed by the included articles. VO_2_ peak - Peak oxygen uptake, HR - Heart rate, LVEF - Left ventricular ejection fraction, QoL - Quality of life

First author, Year	Outcomes addressed									
	VO_2_ peak	HR	LVEF	Cardiac remodeling	Cardiac biomarkers	Vascular function	Blood pressure	Body composition	Adverse events	QoL
Heart failure										
Ellingsen et al. 2017 [14]	I		I	I	I				I	I
Xie et al. 2017 [2]	I	I	I			I	I	I		
Lima et al. 2018 [24]						I	I		I	
Hsu et al. 2019 [23]	I	I	I	I	I					I
Besnier et al. 2019 [22]	I	I	I						I	
Dun et al. 2019 [12]	I	I				I		I	I	
Mueller et al. 2021 [13]	I			I	I				I	I
Heart transplant										
Perrier-Melo et al. 2018 [25]	I	I			I	I	I	I	I	
Nytrøen et al. 2019 [21]	I	I		I	I	I		I	I	I

## Review

Discussion

This section discusses the effects of HIIT among HF patients and HTx recipients. These include, but are not limited to, VO_2_ peak, heart rate, LVEF, cardiac remodeling, cardiac biomarkers, vascular function, blood pressure, body composition, adverse events, and QoL. Based on our research, the previous systematic reviews on this topic have focused on participants either with HF or HTx. Moreover, these reviews explained the outcomes of HIIT that concentrated more on the VO_2_ peak and intervention of greater than four weeks [2,25]. This systematic review found that HIIT is generally superior or similar to other exercise training on VO_2_ peak, heart rate, LVEF, cardiac biomarkers, vascular function, blood pressure, body composition, and adverse events in HF patients. The aforementioned is also true with QoL among HTx recipients. In addition, data collected is inconclusive for cardiac remodeling and QoL among HF patients.

HIIT on Heart Failure Patients

Improvement of VO_2_ peak has been an independent predictor of mortality among HF patients [2,12]. Every 1-mL/kg/min increase in this outcome provided a 58% reduction in five-year mortality, as explained by Hsu et al. [23]. This increase is supported by studies showing an improvement of the VO_2_ peak for up to 20% [22,23]. One study of HIIT did not show a significant difference compared with MCT, but HIIT remained superior to RRE [14]. However, two studies also stated that VO_2_ peak changes were not maintained with a one-year follow-up [13,14]. This outcome could be due to patients exercising HIIT below the prescribed target [14]. This result is why a more adaptable HIIT protocol is recommended to ensure that the patients continue with the program, such as the gradual increase in speed or progression from short HIIT interval to medium, and then long [12,33,31]. In addition, the stability of the patient's disease should also be considered [34]. Thus, the feasibility of HIIT among HF patients should be accounted for to ensure long-term progress. As a result, the potential of HIIT in improving the VO_2_ peak remains the same. However, the structure of the HIIT intervention should still be further analyzed to be suited to this type of patient. 

**Figure 2:**
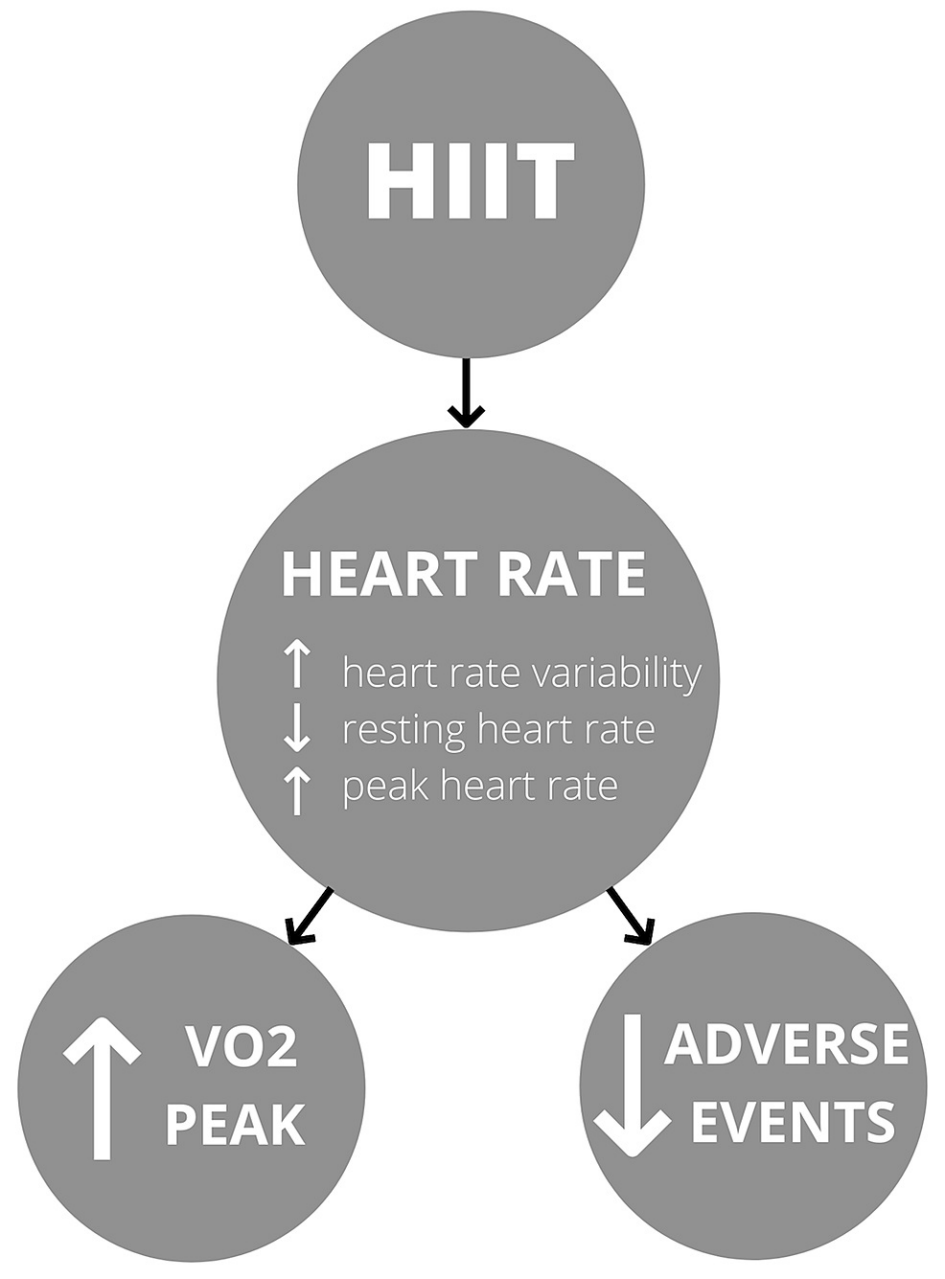
Effects of high-intensity interval training on heart rate and Its effects on VO2 peak and adverse events HIIT - High-intensity interval training, VO_2_ peak - Peak oxygen volume

Heart rate variability, resting, and peak heart rate was included in this review to assess heart rate. A study by Bresnier et al. show that HIIT resulted in more significant heart rate variability from 21.2% to 26.4%, P < 0.001 compared to MICT from 23.1% to 21.9%, P = 0.444. This change has been shown to decrease adverse cardiac events, especially arrhythmias [22]. Peak heart rate showed similar or increased post-HIIT in two studies while resting heart rate decreased significantly for HIIT and MCT [2,22,23]. Isocaloric protocols, however, should be considered in these assessments as to the different needs of each exercise intervention [30]. These changes improve VO_2 _peak, thus enhancing cardiovascular and autonomic nervous system functions [12]. There is a chain of improvement between variables, which possibly shows an association between them. Therefore, heart rate may affect the outcomes of VO_2_ peak and adverse events in HF. The flow chart in Figure 2 summarizes the effects of HIIT on heart rate and heart rate on other outcomes, which the authors illustrated.

The HIIT has shown similar or greater improvements in LVEF, cardiac biomarkers, vascular function, blood pressure, body composition, and adverse events compared with other exercise training. LVEF increased for up to 39.5%, with a higher increase of up to 48.2% among HFrEF patients in training for > 3.5 weeks [2,14,22,23]. Biomarkers only showed a decrease in BNP level among HFrEF patients, while no significant difference was observed with other exercise protocols [13,14,23]. Bresnier et al. add that this decrease reflects a relief of cardiac stress [23]. For vascular function, one HIIT session contributed to an increase in brachial artery diameter of 0.37 ± 0.44 mm [24]. However, this can also be attributed to post-hyperemia. There was no significant difference between groups for flow-mediated dilation [2,24]. This result, however, does not remove HIIT from having the potential to improve vascular function [12]. HF patients with HFrEF are more responsive to HIIT as part of cardiac rehabilitation in improving LVEF and decreasing biomarkers. Furthermore, the findings found in vascular function are in no way definitive because of the short intervention and small sample size.

For interventions greater than four weeks, HIIT has shown no difference in improving blood pressure compared to other exercise protocols [2]. However, a study shows that a single HIIT session can significantly reduce systolic blood pressure (SBP) among HF patients [24]. The results found in SBP may also not be definitive due to the small sample size and the single-session intervention. However, this may still be used in participants who aim for a significant reduction in SBP along with other outcome improvements in cardiac rehabilitation.

For body mass, Xie et al. discuss no significant difference between groups of HIIT and MCT with an MD 0.55 kg, 95% CI −0.52 to 1.62 kg, 𝑝 = 0.31 [2]. Studies also show that HIIT improves total skeletal muscle fiber and mitochondrial function of HF patients and decreases body mass [2,12]. Four studies in this review have shown either no adverse events throughout the exercise protocol of HIIT or no statistical difference between HIIT with other exercise training, even in one-year follow-up [12-14,24]. The HIIT is considered exercise training with a good safety profile among cardiac patients [28]. Thus, HIIT as part of cardiac rehabilitation may decrease body mass among patients with HF with little to no adverse events.

In cardiac remodeling, interventions up to a year did not show a significant difference between HIIT and other exercise training [13,14]. Furthermore, the improvements were not maintained at follow-up after one year [14]. Despite this, a study by Hsu et al. shows a change leading to an improved eight-month survival rate (p = 0.044) in HIIT participants with MDP compared to MDP alone [23]. Thus, combined training may have a greater effect on cardiac remodeling than surmised. However, more extensive studies on this outcome are needed [31]. Nevertheless, this result shows that HIIT may be more effective when combined with MDP than alone as exercise training in cardiac rehabilitation.

For the quality of life among patients, there was no significant difference between HIIT and other interventions [13,14]. However, a study shows that for a selected group of HF patients, those with HFrEF felt better soon after completing HIIT for 12 weeks and even after a one-year follow-up [23]. Furthermore, another study states that HIIT has more remarkable developments in the emotional well-being items for QoL [26]. Mueller et al. contradict these, stating that QoL was significantly higher after a year among those who underwent MCT. The participants included in the study were HFpEF patients [13]. This contradiction requires more studies to fully elucidate the impact of HIIT on QoL among HF patients. Nonetheless, HIIT may influence the QoL of HFrEF patients specifically, even in the long term. 

HIIT on Heart Transplantation Recipients

There were only two studies gathered for HTx. With greater than four weeks of intervention of HIIT, VO_2_ increased up to 15% with HIIT, peak heart rate increased, and resting heart rate decreased, and these changes were observed in the one-year follow-up [21,25]. As aforementioned, these outcomes serve as significant predictors of mortality, as stated by Nytrøen et al. [21,27]. There were also no significant changes in cardiac remodeling in a nine-month HIIT. However, an increase in the left ventricular systolic dimension was observed at the 1-year follow-up [21]. The HIIT positively affected inflammatory biomarkers, vascular function, blood pressure, and QoL, though these changes were not significant. No adverse events related to HIIT were reported in the studies [21,25,33]. There was a positive effect on maximal muscle strength (1 RM) and lean mass maintenance [25]. However, after a one-year follow-up, only the extensor muscle exercise capacity was reported [21]. Thus, the HIIT may significantly improve outcome measures in HTx recipients, especially in VO_2_ peak, heart rate, cardiac remodeling, and body mass. Furthermore, these outcomes may be retained long-term with consistent use of the intervention as part of cardiac rehabilitation.

HIIT on Heart Failure Patients Before and After Heart Transplantation

**Figure 3:**
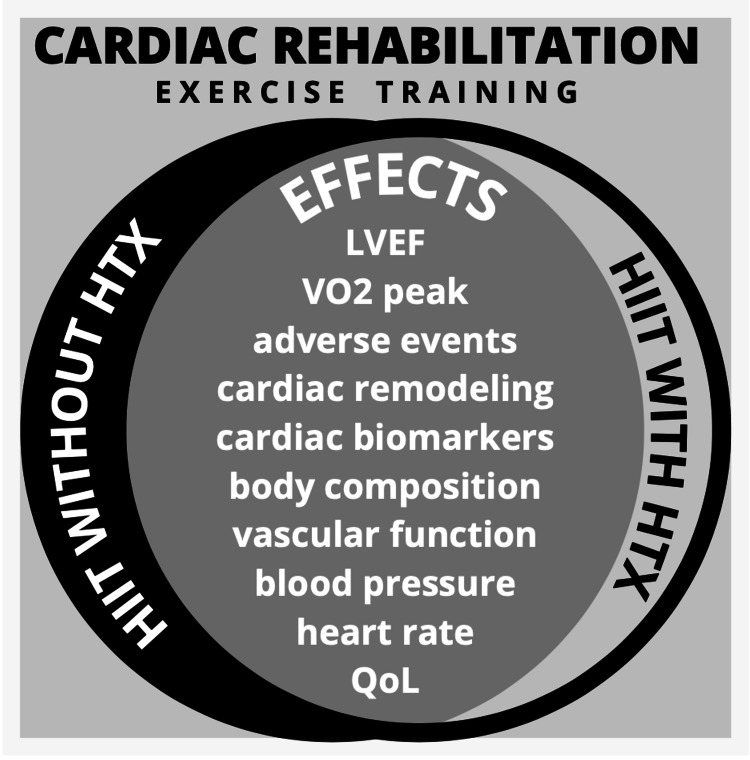
Effects of high-intensity interval training as part of cardiac rehabilitation on heart failure patients before and after heart transplantation HIIT - High-intensity interval training, HTx - Heart transplantation, LVEF - Left ventricular ejection fraction, VO_2_ peak - Peak oxygen volume, QoL - Quality of life

No studies in the review had HF patients who underwent HIIT before and after HTx. However, the results show that HIIT can have positive effects on the outcome variables included in the study for either HF patients or HTx recipients. The investigators of this review summarized the effects of both HF patients with or without HTx undergoing HIIT as exercise training in cardiac rehabilitation. This may affect the recommendation of exercise prescription among these populations, serving as a bridge to greater improvement of the prognosis of HF patients undergoing HTx. These are presented in Figure 3.

Limitations

This review limited the included studies to the English language published in five databases from 2016 to 2021. Grey literature and other databases were also not included. Moreover, the review was also restricted by the heterogeneity of the studies and the varying pharmacology involved. The studies gathered varied in participants: HF patients - HFrEF, HFpEF, or both, and no studies of HIIT on HF patients before and after HTx were found; workouts: cycling or treadmill, and exercise protocols: all lengths of exercise interventions, so long as one session was done, were included in the review. There was no in-depth analysis on the different kinds of intervals - short-, medium-, and long- and the mechanisms resulting in the affected outcomes were not explained.

Furthermore, there was a variation in the total duration of follow-up, and all these factors may lead to inconsistency in conclusion. Therefore, this review recommends RCTs and observational studies conducted with larger sample sizes and longer durations of follow-up either in HIIT alone or with MCT among HF patients, HTx recipients, and HF patients before and after HTx. Furthermore, additional studies are needed to determine which HIIT exercise is better to assure more significant benefits among these patients.

## Conclusions

In conclusion, the studies included in this review show that high-intensity interval training (HIIT) is promising and either similar or superior to other exercise training in cardiac rehabilitation. This assessment is in terms of VO_2_ peak, heart rate, left ventricular ejection fraction (LVEF), cardiac biomarkers, vascular function, blood pressure, body composition, and adverse events among heart failure (HF) patients and heart transplantation (HTx) recipients. In addition, HIIT also has the potential to have positive effects on the outcome variables included in the study for HF patients before and after Htx. However, for cardiac remodeling and QoL of HF patients, data on HIIT effects remain inconclusive. Nevertheless, this outcomes summary of HIIT on HF patients and HTx recipients provides a more concise HIIT recommendation that may be used in cardiac rehabilitation in improving prognosis and management. Future suggestions regarding this study include conducting and adding more studies, especially cohorts, with larger sample sizes and longer durations of follow-up either with HIIT alone or in combination with moderate-continuous training (MCT). These recommendations are made to examine further the outcome measure on HF patients, HTx recipients, and HF patients before and after HTx, and possibly bridge the gap of determining the effects of HIIT on HF patients before and after HTx. Furthermore, HIIT with different durations - short, medium, or long- should be further assessed.
